# Severe myositis of the hip flexors after pre-operative chemoradiation therapy for locally advanced rectal cancer: case report

**DOI:** 10.1186/s12885-016-2269-2

**Published:** 2016-03-22

**Authors:** Matthew M. Florczynski, Michael S. Sanatani, Lauren Mai, Barbara Fisher, Dwight E. Moulin, Jeffrey Cao, Alexander V. Louie, Janet E. Pope, Eric Leung

**Affiliations:** Departments of Oncology, Schulich School of Medicine and Dentistry, Western University, London, ON Canada; Departments of Rheumatology, Schulich School of Medicine and Dentistry, Western University, London, ON Canada; Departments of Neurology, Schulich School of Medicine and Dentistry, Western University, London, ON Canada; Department of Radiation Oncology, Sunnybrook Health Sciences Centre, University of Toronto, Toronto, ON Canada

**Keywords:** Myopathy, Neoadjuvant, Rectal cancer, Radiation recall

## Abstract

**Background:**

The use of neoadjuvant radiation therapy and chemotherapy in the treatment of locally advanced rectal adenocarcinoma has been shown to reduce disease recurrence when combined with surgery and adjuvant chemotherapy. We report a case of a patient who developed a debilitating bilateral myopathy of the hip flexors after successful treatment for rectal cancer. To the best of our knowledge, this is the first such complication from radiation therapy reported in a patient with colorectal cancer. The disproportionate severity of our patient’s myopathy relative to the dose of radiation used also makes this case unique among reports of neuromuscular complications from radiation therapy.

**Case presentation:**

The patient is a 65-year-old male with node negative, high-grade adenocarcinoma of the rectum penetrating through the distal rectal wall. He underwent neoadjuvant concurrent pelvic radiation therapy and capecitabine-based chemotherapy, followed by abdominoperineal resection and post-operative FOLFOX chemotherapy. Five months post-completion of pelvic radiotherapy and 2 months after the completion of adjuvant chemotherapy, he presented with bilateral weakness of the iliopsoas muscles and severe pain radiating to the groin. The patient improved with 40 mg/d of prednisone, which was gradually tapered to 2 mg/d over 6 months, with substantial recovery of muscle strength and elimination of pain.

**Conclusions:**

The timing, presentation and response of our patient’s symptoms to corticosteroids are most consistent with a radiation recall reaction. Radiation recall is a phenomenon whereby previously irradiated tissue becomes vulnerable to toxicity by subsequent systemic therapy and is rarely associated with myopathies. Radiation recall should be considered a potential complication of neoadjuvant radiation therapy for rectal cancer, and for ongoing research into the optimization of treatment for these patients. Severe myopathies caused by radiation recall may be fully reversible with corticosteroid treatment.

## Background

Globally, colorectal cancer (CRC) is the third most common cause of cancer and the fourth leading cause of cancer mortality [1]. Almost one-third of primary CRCs are adenocarcinomas localized to the rectum between its borders at the sigmoid colon and the dentate line. Locally confined, low grade (T1-2) lesions (stage I) can generally be treated with total mesorectal excision (TME) surgery or even local excision, usually sparing the anal sphincter. However, locally advanced T3-4 disease penetrating through the wall of the rectum (stage II) or involving local lymph nodes (stage III) requires aggressive multimodal therapy.

**Fig. 1:**
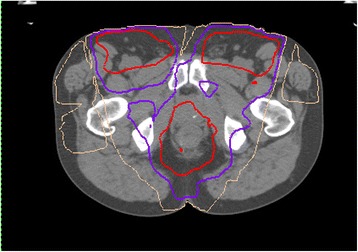
Intensity-modulated radiation plan for pre-operative chemoradiation treatment for locally advanced rectal cancer. Axial image at the level of the proximal thigh. Inguinal nodes were treated based on the low-lying location of the rectal lesion. Radiation dose prescription = 5000 cGy. Isodose lines, red: 95 %, purple: 80 %, beige: 50 %

**Fig. 2:**
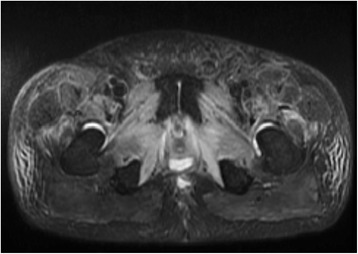
MRI pelvis post-radiation, axial STIR/T2W FSE sequence showing edema of proximal leg muscles

For patients with stage II-III rectal adenocarcinoma, the standard of practice in most of Europe and North America involves three phases of treatment. Treatment begins with a course of pre-operative (*neo*adjuvant) chemotherapy and radiation therapy (CRT), followed by surgical resection of the tumour with sphincter-sparing TME or non-sphincter-sparing surgery, most commonly either a low anterior resection (LAR) or abdominoperineal resection (APR), and post-operative (adjuvant) chemotherapy. The addition of post-operative CRT to surgical management dramatically reduced cancer recurrence and improved survival in patients with stage II-III disease [2, 3]. Neoadjuvant CRT in conjunction with surgery has been shown to confer a further reduction in local disease recurrence and to delay recurrence compared to adjuvant CRT with surgery [4, 5].

In this report, we present a patient who experienced a rare complication following neoadjuvant CRT, surgery, and adjuvant chemotherapy for locally invasive rectal adenocarcinoma. During the adjuvant chemotherapy phase of treatment, our patient developed a bilateral myopathy in his hip flexors that presented with sudden-onset severe pain and debilitating muscle weakness. His condition was successfully managed with high-dose corticosteroids tapered over several months. The severity of this case makes it unique among reports of neuromuscular complications from low doses of radiation therapy (RT); furthermore, this is the first such case resulting from neoadjuvant CRT for locally advanced rectal cancer.

## Case presentation

A 65-year-old male was referred for investigation of a rectal mass. The patient had a 2-year history of painless, bright red bleeding per rectum. During this period he had experienced increased fatigue and one bowel movement per day with deformed, bloody stool. Apart from having a polyp removed 1 year ago, he had no past medical history or chronic medical conditions. The patient’s father had prostate cancer and his mother had ovarian cancer.

Digital rectal examination revealed an ulcerated mass beginning at the anorectal junction, tethered along the rectal canal to the overlying puborectalis muscle. A diagnosis of stage II (T3N0M0) invasive adenocarcinoma was made on biopsy and imaging. Magnetic resonance imaging (MRI) of the pelvis showed that the tumour was located 4.5 cm above the anal verge and had an extramural depth of invasion of 6 mm. It extended approximately 5.3 cm from the distal rectum along the right side of the rectal canal. Coronal views suggested that the mass penetrated the mesorectal fascia. Invasion of the intersphincteric plane could not be excluded. There was no involvement of adjacent lymph nodes or the peritoneal reflection. Computed tomography (CT) of the abdomen and pelvis were negative for metastases.

Treatment consisted of three phases: (1) 5 weeks of neoadjuvant CRT, (2) followed 4 weeks later by APR surgery, and (3) 8 weeks of adjuvant chemotherapy. Pelvic radiation with a total dose of 50 Gy was delivered in 25 fractions (5 days/week over 5 weeks) with a minimum of 10 mV photons. A six-field technique was used (anterior oblique, oblique and posterior oblique fields bilaterally) weighted higher in the right fields (Fig. 1). The maximum dose to the right femoral head was 4950 cGy (mean 3482 cGy) compared to a maximum dose of 4705 cGy (mean 3173 cGy) to the left femoral head. Inguinal nodes were included in the treatment based on the low-lying location of the rectal lesion. Concurrent chemotherapy was administered with capecitabine (3300 mg/day PO) delivered during radiation. Four weeks into neoadjuvant treatment the patient developed hand-foot syndrome so his dose of capecitabine was lowered. One week after completion of CRT, the patient presented with painful, red, ulcerating dermatitis on both sides of the groin with desquamated skin overlying the penis and scrotum, as well as perianal redness and excoriations, all of which healed after management with flamazine and sitz baths. A repeat MRI of the pelvis following neoadjuvant CRT showed that the extent of the tumour along the rectal canal had been reduced to 4.5 cm and the extramural depth of invasion had been reduced to 4 mm.

Radical surgery consisted of APR with mesocortical excision. Post-operative pathology confirmed the diagnosis of stage II (T3a) adenocarcinoma. Macroscopically, a single polypoid mass was present with tumour-free margins and undamaged non-tumoural tissue. Microscopic analysis of the tissue revealed a low grade tumour that did not penetrate the rectal serosa. The smallest tumour-free margin was 2.5 mm and no local node invasion was found. Evidence of tumour response to neoadjuvant treatment was minimal and treatment-related changes in the non-tumoural tissue were not described. Following surgery, adjuvant FOLFOX chemotherapy (5-fluoruracil, leucovorin, oxaliplatin) was delivered IV for eight cycles.

Five months after the completion of pelvic radiotherapy and 2 months after the completion of chemotherapy, the patient presented with severe bilateral leg weakness and pain that required hospitalization. The patient had a 5-week history of increasing, movement-aggravated pain in the inguinal region that radiated to the thighs, and a 3-week history of worsening proximal muscle weakness of the lower extremities. He assumed a stooped, forward-flexed posture as this provided some pain relief. On exam, the patient could only walk for a few steps. There were palpable areas of fullness in the femoral triangle, but no discrete lymphadenopathy. Motor power was less than antigravity in the hip flexors bilaterally, and was independent of pain. The patient was unable to elevate his legs at the hip or flex them by more than 10°. Femoral stretch and straight leg-raise tests severely aggravated pain in the groin and anterior thighs. He had full power in his hip extensors, knees and ankles. His knee reflexes were decreased and ankle reflexes were absent. A CT scan of the pelvis was negative for abscesses. His bone scan did not show abnormal involvement and his MRI of the spine was not suggestive of a radiculopathy or other spinal cord process. Creatine kinase (CK) was within the normal range. The MRI of his pelvis obtained 2 months after the completion of adjuvant chemotherapy was remarkable for edema involving multiple muscle groups of the proximal thighs bilaterally, consistent with an acute myopathic process (Fig. 2). An electromyographic (EMG) study revealed moderate fibrillation potentials, positive sharp waves, and absent recruitable motor units in the iliopsoas muscles, further supporting a diagnosis of bilateral myopathy in this distribution. Needle examination of the abductor longus, vastus medialis and tibialis anterior were unremarkable.

At the time of presentation, the patient was severely debilitated and hospitalized for 3 weeks. His muscle weakness and pain could only be controlled with a high dose of prednisone (40 mg/d PO), which provided substantial symptom relief within 2–3 days. After discharge, the patient was able to walk 4–5 short city blocks without taking a break, but continued to experience some pain at rest. Over the next 16 weeks prednisone was tapered to 2 mg/d. This resulted in an increase in pain and activity limitation over the first 6 weeks, but his condition has been gradually improving since then with reduced doses of prednisone. At last follow-up, he was no longer experiencing pain and had grade 4+/5 power in his hip flexors and grade 4/5 power in his hip adductors bilaterally. He is expected to be weaned off prednisone over the next few months.

## Conclusions

We present the case of a patient who experienced a severe myopathy following treatment for rectal adenocarcinoma. Shortly after the completion of adjuvant chemotherapy, the patient presented with near-complete functional limitation due to muscle weakness and severe pain. His symptoms were localized to the thighs bilaterally, areas that received radiation during neoadjuvant treatment, albeit in doses well within muscle tolerance. Although the patient has yet to reach a complete functional recovery, the strong response of both muscle weakness and pain to gradually tapered doses of corticosteroids is an encouraging sign that this complication of CRT is at least partially reversible.

The cause of our patient’s symptoms is difficult to pinpoint since multiple treatment modalities were used, including RT and two different chemotherapy regimens. Based on our review of comparable cases in the literature, we considered two scenarios by which a myopathic process could have been precipitated in our patient: (1) as a delayed reaction to RT or (2) as a result of additive effects of radiation and chemotherapy causing a radiation recall reaction.

The location of myopathic changes in our patient suggests that RT was a precipitating factor. Peripheral nervous system dysfunction, plexopathies, myelopathies and mononeuropathies are well-documented adverse events following RT, with location and size of the radiation field being pertinent risk factors [6]. Elderly patients or those with poor overall health status have particularly poor outcomes following extended-field RT compared to more focal treatment [7]. RT-induced myopathy in the absence of other neurological findings is extremely rare, most commonly reported in patients with Hodgkin’s lymphomas treated with high-dose mantle field RT [8–12]. Ghosh and Milone recently published a large case series describing 21 patients with radiation-induced myopathy, in which 13 patients were treated for Hodgkin’s lymphoma [9]. Almost half of the patients had severe neck pain and muscle weakness causing head drop. Two patients died during follow-up due to related hypercapnic respiratory failure. Similar to our patient, the majority of these patients had myogenic changes on EMG with fibrillation potentials, and near normal CK levels. Unlike our patient, many of these patients had profound muscle atrophy and muscle contractures, as well as associated neurogenic findings on physical exam, EMG or imaging. Perhaps the most notable discrepancy between previous reports of radiation-induced myopathies and our case is the onset latency of myopathic changes, typically occurring years or decades after RT.

A second possible explanation is that chemotherapy exacerbated tissue injury caused by RT in our patient. Such a complication is known as radiation recall, a rare phenomenon whereby previously irradiated tissue becomes vulnerable to toxicity by subsequent systemic therapy [13]. Anti-cancer drugs are the most common causes of radiation recall, but other precipitating agents include antibiotics, anti-tuberculosis agents, and simvastatin [14]. Radiation recall appears to be distinct from radiosensitization, a much more common phenomenon thought to last for up to 1 week after the completion of RT. Radiation recall most commonly manifests as dermatitis in previously irradiated skin [15], but it can also manifest as myositis [16–19], enterocolitis [20], or pneumonitis [21, 22].

There are several factors supporting radiation recall as the cause of the complication seen in our patient. Although a variety of anti-neoplastic drugs are associated with radiation recall [14], anti-metabolites are among the most common precipitating agents. This drug class includes capecitabine and 5-fluorouracil (5-FU), both of which were used to treat our patient. Consistent with the presentation in our patient, radiation recall myositis typically occurs within a few months of initiation of chemotherapy and evidence of soft tissue edema on MRI appears to be replicated finding [16–18]. Since any tissue injury caused by RT in these patients remains subclinical until the initiation of chemotherapy, high doses of RT are not required to induce radiation recall. Indeed, a comparable dose of RT (54 Gy, 28 fractions) was used in a patient who developed a gemcitabine-induced myopathy of the rectus abdominus muscles 5 months after CRT for pancreatic cancer [17]. Grover et al. recently described a case in which a patient treated with RT, gemcitabine and carboplatin developed myositis in the left shoulder and hip, with corresponding tissue edema on MRI, 4 weeks after completion of RT [18]. Both foci of this patient’s metastatic adenocarcinoma were treated with lower total doses of 30 Gy in 10 fractions and the myositis was relatively mild and self-limited. Severe radiation recall myositis has been described in at least one report of a 14-year-old girl who received neoadjuvant CRT prior to surgery and post-operative gemcitabine for a synovial carcinoma of the forearm [16]. She developed severe weakness, pain and tissue edema in her forearm after the cessation of gemcitabine. This precipitated a compartment syndrome that lasted for 1 year. Interestingly, this case was also responsive to corticosteroids and gradually resolved with tapering. Other reports suggest that the response of radiation recall to steroids is variable [23], and withdrawal of the precipitating drug may be enough to resolve mild myopathies [17, 18]. In summary, previous reports of radiation recall are consistent with the onset, dose of RT, presentation and response to treatment observed in our patient.

There are at least a couple of noteworthy cautions regarding the interpretation of our findings. Firstly, a tissue sample of the affected muscle in our patient was not obtained, limiting our ability to compare this case with previous biopsy-proven cases of radiation-induced myopathies [24]. However, the pathological analysis of our surgical specimen revealed relatively preserved structural integrity of the non-invaded tissues surrounding the tumour. Since these undamaged tissues were focally irradiated, myonecrosis in the patient’s adjacent hip flexors as a result of CRT would not be expected. Secondly, it is possible that causes unrelated to radiation exposure precipitated the myopathy in our patient. Myopathic changes due to chemotherapy have been observed in the context of oxaliplatin-induced peripheral neuropathies [25] and capecitabine-associated dermatomyositis [26]. While our patient did develop peripheral neuropathy during oxaliplatin-based (FOLFOX) adjuvant chemotherapy, this had a period of onset distinct from his myopathy. A chemotherapy-only phenomenon in our patient is unlikely due to the localized nature of our findings corresponding to regions of previously irradiated tissue.

We have presented a case of an extremely rare complication of RT that is notable both for its severity and location of presentation. This is the first time that this complication has been reported in a patient treated for CRC. The use of neoadjuvant CRT in patients with locally advanced rectal adenocarcinoma is a recently adopted standard of care that has decreased the likelihood of cancer recurrence in this patient population [4, 5]. Our findings are particularly relevant in light of the fact that optimization of CRT has been a focus of recent and ongoing clinical trials [27–29]. 5-FU is the most commonly used neoadjuvant chemotherapeutic agent in this patient population. Capecitabine has demonstrated similar efficacy to 5-FU and was chosen for our patient due to the convenience of administration in its oral form [27, 28].

So far, data from clinical trials have shown that neoadjuvant CRT is well tolerated by patients with rectal cancer. In patients receiving 5-FU-based CRT with 50.4 Gy of total radiation, the most common acute complications were dermatologic (27–40 %), diarrhea (12–18 %) and hematologic (6–8 %) [4]. The most common long-term complications were gastrointestinal (diarrhea, small bowel obstruction; 9–15 %), strictures at the site of large bowel anastomosis (4–12 %) and bladder dysfunction (2–4 %) over 5 years of follow-up. Although these complications are difficult to link directly to chemotherapy or radiotherapy, the use of neoadjuvant CRT significantly reduced both acute and chronic complications compared to adjuvant CRT. Pre-operative administration of chemotherapy and radiation thus appears to reduce toxicities related to these treatments.

Our case report contributes to a growing body of data for a complication of RT that remains poorly understood. Although we cannot clearly ascertain whether our patient’s symptoms were a direct result of RT or caused by radiation recall, it is important to consider both complications within the realm of possibilities in patients receiving the current 3-phase treatment for locally advanced rectal cancer. Mild to moderate cases of radiation-induced myopathy may be underreported due to loss of patients to follow-up. Radiation recall may also be underreported, particularly mild cases that disappear following withdrawal of chemotherapy. Optimal doses of RT and chemotherapy should continue to be sought in order to minimize complications. It has been suggested that radiation recall can be prevented by allowing a sufficient time interval between neoadjuvant RT and adjuvant chemotherapy [14]; however, at least one case of radiation recall was reported 21 years after RT, so the appropriate time interval may vary on an individual basis [19]. While prevention of myopathic complications in the cancer population may be challenging so long as these phenomena remain poorly understood, our report demonstrates encouraging evidence of the effectiveness of corticosteroid treatment for severe cases.

## Consent

Written informed consent was obtained from the patient for publication of this case report and any accompanying images. A copy of the written consent is available for review by the editor of this journal.

## Abbreviations

5-FU: 5-fluorouracil
APR: Abdominoperineal resection
CK: Creatine kinase
CRC: Colorectal cancer
CRT: Chemotherapy and radiation therapy
EMG: Elecromyography
FOLFOX: 5-fluorouracil leucovorin, oxaliplatin
MRI: Magnetic resonance imaging
RT: Radiation therapy
TME: Total mesorectal excision
